# Functional Analysis of BmHemolin in the Immune Defense of Silkworms

**DOI:** 10.3390/insects16080778

**Published:** 2025-07-29

**Authors:** Long He, Lijing Liu, Huawei Liu, Xin Tang, Yide Meng, Hui Xie, Lin Zhu, Qingyou Xia, Ping Zhao

**Affiliations:** 1 Biological Science Research Center, Southwest University, Chongqing 400715, China; 2 Chongqing Key Laboratory of Innovative Chinese Medicine and Health Intervention, Chongqing Academy of Chinese Materia Medica, Chongqing University of Traditional Chinese Medicine, Chongqing 400065, China

**Keywords:** BmHemolin, immune defense, artificial diet, CRISPR/Cas9, antimicrobial peptides, phagocytosis-related factors

## Abstract

*Hemolin* has been identified as a crucial immune gene in insect immune defense. The silkworm is susceptible to infections by pathogenic microorganisms when reared on artificial diets. In this study, through comparative analysis of the expression patterns of BmHemolin in silkworms fed on mulberry leaves and artificial diets, we found that the expression of BmHemolin was significantly upregulated in silkworms reared on artificial diets, and this upregulation was highly likely induced by pathogenic microorganisms. Further interaction analysis revealed that BmHemolin could bind to pathogenic microorganisms and form aggregates. Meanwhile, BmHemolin enhanced the melanization and aggregation of hemocytes. Subsequent in vitro antibacterial experiments showed that BmHemolin had the ability to inhibit the growth of *Escherichia coli*. In vivo clearance experiments demonstrated that BmHemolin facilitated the clearance of pathogens in the body. Moreover, CRISPR/Cas9-mediated knockout of the BmHemolin gene led to the downregulation of antimicrobial peptides and phagocytosis-related factors, while an excess of BmHemolin could enhance the expression of these genes, thereby improving the silkworm’s immune resistance to *Enterococcus mundtii* and increasing survival rates. In summary, our research demonstrates that BmHemolin played a pivotal role in both humoral and cellular immunity in the silkworm, thereby defending against pathogen invasion.

## Simple Summary

*Hemolin*, as a crucial immune gene, plays a pivotal role in the immune defense of insects. Understanding its mechanism of action is of great importance for the immunity of insects. This study focuses on investigating the immune function of the BmHemolin gene in the silkworm (*Bombyx mori*), utilizing CRISPR/Cas9 gene editing technology to explore its mechanism of action. The research demonstrates that BmHemolin participates in the cellular immunity of the silkworm by promoting hemocytes melanization and aggregation, and by regulating the expression of cellular phagocytosis-related factors such as *Ced6*. Additionally, it is involved in the humoral immunity of the silkworm by modulating the expression of antimicrobial peptides.

## 1. Introduction

The insect possesses a complex and efficient innate immune system that effectively combats infections caused by pathogens. This innate immune system comprises two major branches: humoral immunity and cellular immunity [1]. Humoral immunity encompasses the production of melanin, and the induction of antimicrobial peptides (AMPs) synthesis [2,3,4]. AMPs play a primary role in bactericidal activity, which are secreted into the hemolymph to exert bactericidal effects. The synthesis of AMPs is mainly regulated by Toll and IMD signaling pathways [1,5,6]. Pattern recognition receptors (PRRs) recognize pathogen-associated molecular patterns (PAMPs), initiating a proteolytic cascade that processes the precursor cytokine pro-Spätzle into its mature ligand [7,8]. The mature Spätzle binds to Toll receptors, activating the NF-κB signaling pathway [9,10], which induces the expression of AMPs. Cellular immunity is mediated by hemocytes, involving processes such as phagocytosis, agglutination, nodule formation, and encapsulation [11,12]. Studies have shown that cytoskeletal proteins, adhesion molecules, and signal transduction enzymes are cooperatively involved in the process of phagocytosis of bacteria by hemocytes [13].

Research has shown that the innate immune system of insects utilizes numerous PRRs to recognize PAMPs, thereby triggering various immune defense mechanisms [14,15,16,17]. PRRs that have been proven include peptidoglycan recognition proteins (PGRPs), β-1,3-glucan recognition proteins (βGRPs), C-type lectins (CTLs) and so on [18,19]. In *Manduca sexta*, Hemolin was identified as a novel PRR that binds to Lipopolysaccharide (LPS) and Lipoteichoic Acid (LTA) to participate in immune defense. Hemolin can bind to the lipid A portion of LPS and is partially inhibited by phosphate and calcium [20,21]. Hemolin belongs to the Immunoglobulin Superfamily (IgSF) and possesses four immunoglobulin-like domains arranged in a horseshoe shape, showing high homology with neural adhesion molecules in both insects and vertebrates [22,23]. Hemolin has been identified in various insects, such as *Hyalophora cecropia*, *Manduca sexta*, and *Antheraea pernyi*. Initially, in *H. cecropia*, Hemolin has been identified as a key component of the immune defense protein complex, which is capable of binding to bacterial surfaces as well as to hemocytes membranes, thereby promoting the occurrence of phagocytosis [23,24]. In *M. sexta*, Hemolin not only functions as a pattern recognition receptor but also mediates microbial agglutination by binding to bacterial and yeast surfaces [21]. In addition, Hemolin is involved in the regulation of humoral immunity, as demonstrated in *A. pernyi* by interfering with *ApHemolin*, proving its participation in the Toll and IMD signaling pathways to regulate AMPs expression [25]. Although the importance of *Hemolin* in insect immune defense has been preliminarily confirmed, its complete mechanism still requires further investigation. Similarly to most insects, the silkworm possesses a complex innate immune system. Its PRRs can specifically recognize pathogenic microorganisms and regulate immune responses through signaling pathways such as Toll, IMD, and JAK/STAT [4]. The functions of various PRRs, including *BmPGRP* and *BmβGRP* have been reported [26,27,28,29]. However, the role of BmHemolin as a PRR in silkworms remains uncharacterized.

As an ideal model for studying the insect immune system, the rearing of silkworms has long been constrained [30]. In recent years, the artificial diet feeding of silkworms has been widely promoted as it overcomes the seasonal limitations of mulberry leaves and reduces labor demands [31]. However, silkworms fed with artificial diets are more susceptible to infections by pathogenic microorganisms such as *Enterococcus mundtii (E. mundtii)*, leading to frequent occurrences of bacterial diseases [32,33]. In our preliminary comparative transcriptome analysis, we observed a significant upregulation of *BmHemolin* transcription levels in silkworms reared on artificial diet, which we hypothesize is due to infection by pathogenic microorganisms such as *E. mundtii*. This study aims to investigate the function of BmHemolin as a PRR and its role in the artificial diet rearing of silkworms.

In this research, we compared and detected the expression of BmHemolin in silkworms fed with mulberry leaves and artificial diets, revealing the interaction between BmHemolin and pathogenic microorganisms. Using the CRISPR/Cas9 knockout system, we successfully knocked out the *BmHemolin* gene and investigated its regulatory effects on AMPs and phagocytosis-related factors. Simultaneously, the impact of excessive BmHemolin on the immune defense of silkworms was analyzed. The findings have enhanced our understanding of the function of the *Hemolin* gene and provide a theoretical basis for the prevention and control of bacterial diseases caused by pathogenic microorganisms in silkworms reared on artificial diets.

## 2. Materials and Methods

### 2.1. Strains, Insects, and Diets

The strains utilized in this study were preserved and supplied by the Biological Science Research Center at Southwest University, China. These included Gram-positive bacteria (G+) such as *E. mundtii* and *Micrococcus luteus* (*M. luteus*), Gram-negative bacteria (G-) such as *Escherichia coli* (*E. coli*) and *Pseudomonas aeruginosa* (*P. aeruginosa*), as well as the fungus *Saccharomyces cerevisiae* (*S. cerevisiae*). Silkworms were reared under controlled environmental conditions of 25 ± 2 °C, 85% relative humidity, and a photoperiod of 12 h light and 12 h dark, using either fresh mulberry leaves (M) or an artificial diet (A). Both the fresh mulberry leaves and the artificial diet were sourced from the Biological Science Research Center at Southwest University. The primary components of the artificial diet consisted of mulberry leaf powder, defatted soybean meal, corn flour, binders, and a mixture of vitamins and inorganic nutrients [31].

### 2.2. Extraction of RNA and Proteins

Silkworm tissues were dissected in pre-chilled phosphate-buffered saline (PBS, 10 mM sodium phosphate, 0.15 M NaCl, pH 7.4) and homogenized using a disposable grinder (Shanghai Sangon, Shanghai, China). For RNA, the TRIzol reagent (Shanghai Sangon, Shanghai, China) was used to extract RNA from the homogenized tissues. For proteins, RIPA buffer (Shanghai Sangon, Shanghai, China) was added to the homogenized tissues, followed by oscillation at 4 °C for 30 min to lyse and extract proteins. The protein concentration was determined using a BCA protein assay kit (Beyotime Biotechnology, Shanghai, China).

### 2.3. cDNA Generation, and Real-Time Quantitative PCR (RT-qPCR)

Complementary DNA (cDNA) was synthesized according to the manufacturer’s instructions for the StarScript III All-in-one RT Mix with gDNA Remover (CenStar, Beijing, China). The cDNA concentration was measured and diluted to 200 ng/µL. The RT-qPCR experiment was performed using the qTOWER 2.2 real-time PCR thermal cycler system (Analytik Jena, Thuringia, Germany) in conjunction with SYBR qPCR SuperMix Plus (Novoprotein, Nanjing, China), strictly following the manufacturer’s guidelines. The internal control utilized was the reference gene *BmActin*, and the relative expression levels for the target genes were determined via the 2^−ΔΔCt^ method [34]. Comprehensive details about the quantitative primers can be found in Table S1.

### 2.4. Expression and Purification of the BmHemolin Recombinant Protein

Primers were designed (Table S1) to clone the *BmHemolin* gene fragment. Subsequently, the pET28a-*BmHemolin* prokaryotic expression vector was constructed. This vector was transformed into *E. coli* BL21 (DE3) cells, and positive colonies were screened by sequencing. The positive colonies were then introduced into LB liquid medium with 50 μg/mL of kanamycin and incubated at 37 °C while shaking at 220 rpm. The optical density of the bacterial culture was measured at 600 nm (OD600) using a microplate reader. Upon reaching an OD600 of 0.6, isopropyl-β-D-thiogalactoside (IPTG) was introduced into the culture to attain a final concentration of 0.1 mM. The culture was then continuously shaken at 16 °C and 220 rpm for 20 h. After incubation, the bacterial pellet was collected by centrifugation at 12,000 rpm for 30 min and lysed by sonication on ice. The soluble fraction was collected for analysis. The purification of all 6 × His-tagged recombinant proteins was performed according to the manufacturer’s guidelines, using the Ni-NTA 6FF Sefinose (TM) Resin Kit (Sangon, Shanghai, China) to purify the BmHemolin recombinant protein. The size and purity of the purified protein were assessed by sodium dodecyl sulfate-polyacrylamide gel electrophoresis (SDS-PAGE) and Western blot analysis.

### 2.5. SDS-PAGE and Western Blot Analysis

Protein samples were mixed with 5 × SDS loading buffer at a ratio of 1:5, heated at 100 °C for 10 min, and subsequently separated by SDS-PAGE. Finally, separated proteins in gel were analyzed through Coomassie Blue staining. In the Western blot process, proteins were transferred onto a PVDF membrane, which was then blocked with a 5% skimmed milk solution prepared in TBST (10 mM Tris-HCl, 150 mM NaCl, 0.1% Tween 20, PH 7.4) at 37 °C for 2 h. Subsequently, the membrane was exposed to BmHemolin antibody at a dilution of 1:2500 (in 1% skimmed milk/TBST) at the same temperature for 1 to 2 h. After washing with TBST, the membrane was treated with HRP-conjugated goat anti-rabbit IgG (1:10,000, purchased from Beyotime, Shanghai, China) at 37 °C for 1 h. Signal visualization was conducted using SuperSignal West Femto Maximum Sensitivity Substrate (Thermo, Waltham, MA, USA) in combination with the ChemiScope 3400 Mini imaging system (Clinx Science, Shanghai, China).

### 2.6. Microbial Challenge

The cultures of *E. mundtii*, *E. coli*, and *S. cerevisiae* were prepared overnight and then centrifuged at 4000 rpm, discarding the supernatant. The bacterial cells were washed with PBS. The concentration of bacterial cells was adjusted to 1 × 10^8^ CFU/mL using PBS. A 5 µL volume of the diluted bacterial suspension was injected into 3rd day of 5th larvae, with an equal volume of PBS injected group serving as the negative control, with 10 larvae per group. Samples of different tissues were collected on ice at 3, 6, 9, and 12 h post-injection. Total RNA was extracted from each tissue, and the mRNA levels of the *BmHemolin* gene were assessed by RT-qPCR. Additionally, cell-free hemolymph samples were collected at 4, 8, and 12 h post-injection of *E. mundtii*, and the expression levels of BmHemolin protein were detected by Western blot analysis.

### 2.7. Microbial Binding and Agglutination Assays

A total of 1 mL of overnight cultures of *E. mundtii*, *M. luteus*, *E. coli*, *P. aeruginosa*, and *S. cerevisiae* were centrifuged at 4000 rpm. The bacterial pellets were washed three times with PBS and resuspended in PBST (containing 0.02% Tween-20). Subsequently, BmHemolin recombinant protein was added at a concentration of 0.5 mg/mL in the absence of calcium ions, followed by rotation and incubation at room temperature for 3 h. The samples were then centrifuged at 12,000 rpm, and the bacterial pellets were washed three additional times with PBST. The supernatant and pellet from the final wash were utilized for Western blot analysis. The pathogen and PBST buffer incubated without BmHemolin recombinant protein served as the negative control, while PBST containing BmHemolin recombinant protein was designated as the positive control.

*E. mundtii*, *M. luteus*, *E. coli*, and *P. aeruginosa* labeled with isothiocyanate were suspended in Tris-HCl buffer (50 mM NaCl, 20 mM Tris-HCl, pH 8.0) and adjusted to a final concentration of 1 × 10^9^ CFU/mL. BmHemolin recombinant protein was added to the bacterial suspension at a final concentration of 0.5 mg/mL, with or without the addition of 10 mM calcium ions, using bovine serum albumin (BSA) as a control. For *S. cerevisiae*, only BmHemolin recombinant protein was added, with BSA serving as the control. Following incubation at 37 °C for 1 h, microbial aggregation in each group was observed.

### 2.8. Melanization and Pathogen Growth Inhibition Assay

The in vitro melanization assay was slightly modified according to the method of Dong Z et al. [35]. Ni-NTA agarose beads (5 μL) were incubated with BmHemolin recombinant protein at 4 °C overnight and then washed with PBS. The hemolymph from fifth-instar silkworms was combined with an equivalent volume of Grace’s medium (which consists of 10% PBS, 0.1% double antibiotics, and 200 μL of 10 mM PTU) and subsequently placed into a 48-well plate that had been pre-coated with 1% agarose, allowing it to stand at room temperature for 15 min. The experimental group was treated with BmHemolin-coated Ni-NTA agarose beads, while the control group was treated with uncoated Ni-NTA agarose beads, and incubated at 27 °C. Microscopic observations were made after 6 h and 24 h. In the blocking experiment, the BmHemolin antibody was co-incubated with Ni-NTA agarose beads coated with BmHemolin recombinant protein at 4 °C, followed by repeating the aforementioned steps, For each experimental group, melanization and hemocytes aggregation were examined in three replicate wells, and the percentage of the melanized area relative to the entire field of view was quantified using ImageJ version 1.54g software.

Twenty microliters of overnight cultures of *E. mundtii*, *M. luteus*, *E. coli*, and *P. aeruginosa* were inoculated into 4 mL of fresh LB medium. In a 96-well plate, the pathogen solution and BmHemolin recombinant protein (final concentration 0.25 or 0.5 mg/mL) were added to each well, with chloramphenicol (50 μg/mL) as the positive control. *S. cerevisiae* was diluted to 1 × 10^5^ CFU/mL, and each well was added with bacterial suspension, PDB medium, and BmHemolin recombinant protein (final concentration 0.25 or 0.5 mg/mL). The positive control was EDTA (10 mM), and the negative control was Tris-HCl buffer. Each group was set up in triplicate. Bacteria were grown at a temperature of 37 °C with a shaking speed of 55 rpm, and OD600 was recorded hourly over a period of 6 h. In contrast, fungi were cultivated at 28 °C and a shaking speed of 45 rpm, and OD600 was recorded every 12 h over a period of 72 h

### 2.9. CRISPR/Cas9-Mediated Mutation and Homozygote Screening

A sgRNA specific to the CRISPR/Cas9 system was designed targeting the second exon region of the *BmHemolin* gene using the online prediction tool (https://cctop.cos.uni-heidelberg.de/index.html; accessed on 28 September 2023). The piggyBac-[3xp3-EGFP-sv40-U6-*BmHemolin* gRNA] vector was subsequently constructed. This vector was then mixed with a piggyBac plasmid encoding the piggyBac transposase in a 1:1 ratio and microinjected into silkworm eggs (G_0_). Following the microinjection, the eggs were incubated until hatching. The hatched larvae were reared under standard conditions until reaching the adult stage. G_0_ adult individuals were either self-crossed or mated with wild-type (WT) adult individuals to produce G_1_ individuals. Positive G_1_ individuals exhibiting green fluorescence were screened and crossed with Cas9 transgenic individuals to generate chimeric G_2_ individuals. Subsequently, G_2_ adults displaying effective editing forms were crossed with WT adults to produce G_3_ individuals with a single editing form. The antennae of each G_3_ adult were clipped to extract genomic DNA for genotyping. G_3_ adults with identical effective editing genotypes were crossed to obtain homozygotes. The selected knockout homozygotes were designated as the *KO-BmHemolin* strain. Complete tissues were collected from adult-stage WT and *KO-BmHemolin* individuals, and the expression of the BmHemolin protein in both WT and *KO-BmHemolin* individuals was evaluated using Western blot analysis [36].

### 2.10. Microbial Clearance Assay In Vivo

Ten microliters of *E. mundtii* suspension (5 × 10^6^ CFU/mL) was injected into the hemolymph of 3rd day of 5th instar *KO-BmHemolin* and WT silkworm larvae. Hemolymph was collected 3 h later, and hemolymph (50 μL) was mixed with 450 μL of PBS. Diluted hemolymph (50 μL) was then plated onto *E. mundtii* chromogenic solid medium, which was incubated at 37 °C. After 36 h of incubation, the colonies (which appeared purple) were observed and counted. Each treatment group consisted of 10 larvae. Further, a mixture of 10 μL *E. mundtii* (5 × 10^6^ CFU/mL) with BmHemolin recombinant protein (0.5 mg/mL) was injected into 3rd day of 5th instar WT silkworm larvae. Silkworm larvae injected solely with *E. mundtii* served as the control. Hemolymph was collected 3 h later, followed by repeating the aforementioned steps.

### 2.11. Detection of Antimicrobial Peptides and Phagocytic Factors

The immune challenge was performed by injecting *E. mundtii*, where 5 μL of bacterial suspension (1 × 10^8^ CFU/mL in PBS) was injected into 3rd day of 5th instar larva of WT silkworms, with WT silkworm larvae injected with PBS as the control. Twelve h post-injection, the fat body and hemocytes were collected. The expression of AMPs in the fat body, such as *Defensin2*, *Attacin1*, *Moricin2*, *CecropinD*, *Gloverin4*, and *Lebocin1/2*, as well as the expression of phagocytosis-related factors in hemocytes, such as *Ced6*, *Actin A1*, and *TetraspainE*, were detected by RT-qPCR. Subsequently, five microliters of bacterial suspension (1 × 10^8^ CFU/mL in PBS) was injected into the hemolymph of 3rd day of 5th instar *KO-BmHemolin* and WT silkworm larvae, with 10 larvae per group. After injection, fat bodies and hemocytes were collected at 8 and 12 h, respectively, and the expression of AMPs in the fat bodies and the expression of phagocytosis-related factors in the hemocytes were detected by RT-qPCR.

A mixture of 10 μL containing *E. mundtii* (5 × 10^7^ CFU/mL) and BmHemolin recombinant protein (10 μg) was injected into 3rd day of 5th instar larva, while the control group was injected with *E. mundtii* only, with 10 larvae per group. The fat body and hemocytes were collected at 8 h and 12 h post-injection, and the expression of AMPs and phagocytosis-related genes was detected by RT-qPCR.

### 2.12. Statistics of Survival Rate

To further investigate the impact of BmHemolin on the survival of silkworm larvae under attack by *E. mundtii*, we conducted a survival analysis. First, we injected *E. mundtii* (10 μL, 1 × 10^8^ CFU/mL) into WT and *KO-BmHemolin* individuals on day 0 of the 5th instar. Subsequently, we collected silkworm larvae on day 0 of the 5th instar from the mulberry leaf-fed group (M) and the artificial diet-fed group (A), and injected them with 10 μL of a mixed solution containing *E. mundtii* (1 × 10^8^ CFU/mL) and BmHemolin recombinant protein (0.5 mg/mL). Larvae injected solely with *E. mundtii* served as the control group. The survival rate was monitored every 24 h, and the survival rates from day 1 to day 7 were recorded.

## 3. Results

### 3.1. BmHemolin Expression Was Strongly Induced in Silkworms Reared on Artificial Diet

**Figure 1:**
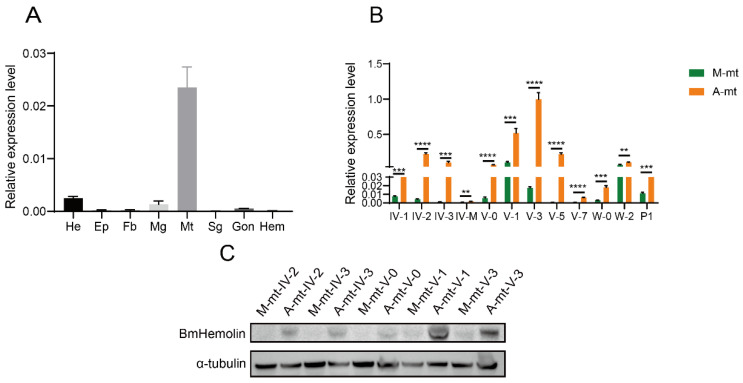
Expression profile analysis of BmHemolin in silkworms reared on mulberry leaves and artificial diet. (**A**) Tissue expression profile analysis of BmHemolin in silkworms reared on mulberry leaves. He: Head; EP: Epidermis; Fb: Fat body; Mg: Midgut; Mt: Malpighian tubules; Sg: Silk gland; Gon: Gonad; Hem: Hemocytes. (**B**,**C**) BmHemolin expression in the Malpighian tubules of silkworms fed with mulberry leaves (M-mt) and artificial diet (A-mt) was compared by RT-qPCR and WB. IV-1 to IV-3: 1st day to 3rd day of 4th instar; IV-M: 4th instar molting; V-0 to V-7: 0 h to 7th day of 5th instar; W-0 and W-2: 0 h to 2nd day after wandering; P1: 1st day after pupa formation.A-mt and M-mt: Malpighian tubules of silkworms fed with artificial diets and mulberry leaves. Error bars represent mean ± SD (*n* = 3), statistically significant differences are indicated as follows: ** *p* < 0.01, *** *p* < 0.001, **** *p* < 0.0001.

RT-qPCR was used to detect and analyze the expression level of *BmHemolin* genes on 3rd day of 5th instar larvae fed with mulberry leaves. The results showed that *BmHemolin* was detected in Head, Midgut, and Malpighian tubules, with the highest expression in Malpighian tubules (Figure 1A). Further analysis of expression of BmHemolin in the Malpighian tubules of silkworms revealed that, compared to silkworms fed with mulberry leaves, both the transcriptional and protein levels of the *BmHemolin* gene were significantly increased in the Malpighian tubules of silkworms fed with artificial diets (Figure 1B,C). The above results indicate that BmHemolin may be involved in the immune defense of silkworms reared on artificial diet.

### 3.2. Pathogens Can Induce Upregulation of BmHemolin Gene Expression

**Figure 2:**
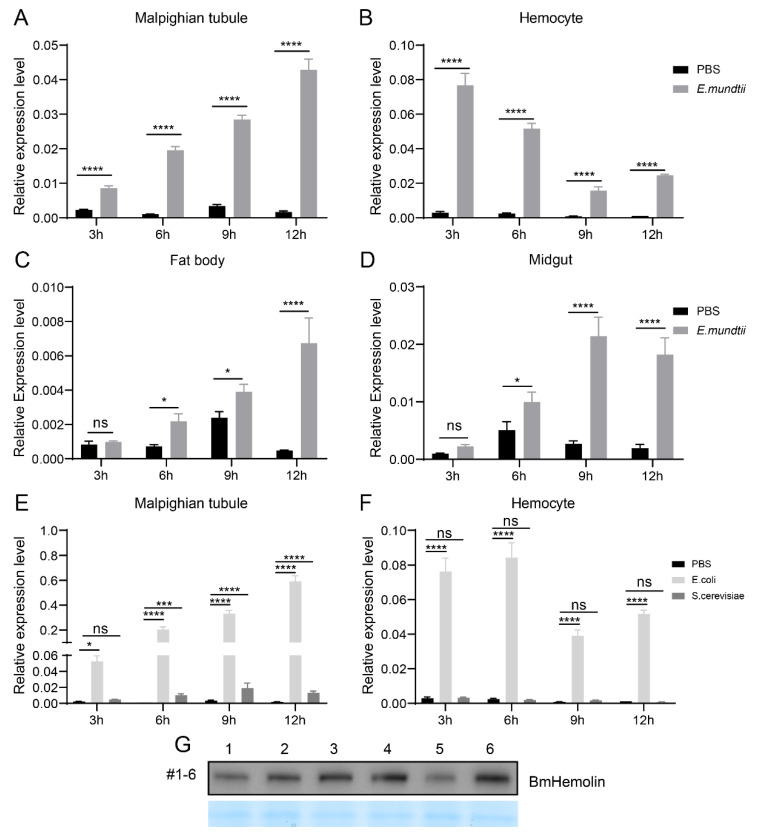
Expression pattern of BmHemolin induced by bacteria. (**A**–**D**) *BmHemolin* mRNA expression was detected at differen time points (3, 6, 9, and 12 h) after injection of *E. mundtii* in Malpighian tubules (**A**); Hemocytes (**B**); Fat body (**C**) and Midgut (**D**). (**E**,**F**) *BmHemolin* mRNA expression levels were detected at different time points (3, 6, 9, and 12 h) after injection of *E. coli* and *S. cerevisiae* in Malpighian tubules (**E**); hemocytes (**F**). Error bars represent mean ± SD (*n* = 3), statistically significant differences are indicated as follows: * *p* < 0.05, *** *p* < 0.001, **** *p* < 0.0001, ns indicates no significance. (**G**) The changes in BmHemolin protein levels in the cell-free plasma after *E. mundtii* injection. Lanes 1, 3, 5: plasma of 5th instar larvae at 4, 8, and 12 h after PBS injection; Lanes 2, 4, 6: plasma of 5th instar larvae at 4, 8, and 12 h after *E. mundtii* injection.

In order to analyze the response of *BmHemolin* gene to pathogenic microorganisms, *E. mundtii*, *E. coli*, and *S. cerevisiae* were used to treat 3rd day of 5th instar larvae, then the immune-related tissues were collected for expression analysis. The results showed that compared to injection with PBS, the expression levels of *BmHemolin* in multiple tissues of silkworms exhibited varying degrees of upregulation after injection with pathogenic microorganisms. In Malpighian tubules, the expression of *BmHemolin* was significantly induced by *E. mundtii*, *E. coli* and *S. cerevisiae* within 3 to 12 h (Figure 2A,E) and the expression of *BmHemolin* increased over time. In Hemocytes, the expression of *BmHemolin* was significantly induced by *E. mundtii* and *E. coli* within 3 to 12 h, but not by *S. cerevisiae* (Figure 2B,F). The expression levels of the *BmHemolin* gene was further detected in the Fat body and Midgut after *E. mundtii* injection. The result showed that there was no significant change in *BmHemolin* gene expression within the first 3 h post-injection, but it was significantly upregulated between 3 and 12 h (Figure 2C,D). Strangely, after the injection of pathogenic microorganisms, *BmHemolin* gradually increases over time in most tissues, but in hemocytes, it initially rises sharply and then declines. A possible explanation is that hemocytes, as the primary executors of the innate immunity in silkworms, first come into contact with the pathogenic microorganisms after they are injected into the silkworm’s hemolymph. *BmHemolin* may be rapidly activated in the early stages of infection and subsequently downregulated to avoid excessive immune responses causing damage to the organism. The Malpighian tubules, Fat body, and Midgut might delay the activation of *BmHemolin* through immune signals transmitted by hemocytes to cope with potential pathogen spread or tissue repair. Further analysis using Western blot revealed that, compared to injection with PBS, the protein level of BmHemolin in the hemolymph was significantly upregulated at 4, 8, and 12 h post-injection with *E. mundtii* (Figure 2G). The above results indicate that BmHemolin can respond to the induction of pathogenic microorganisms.

### 3.3. BmHemolin Can Bind and Agglutinate Pathogenic Microorganisms

**Figure 3:**
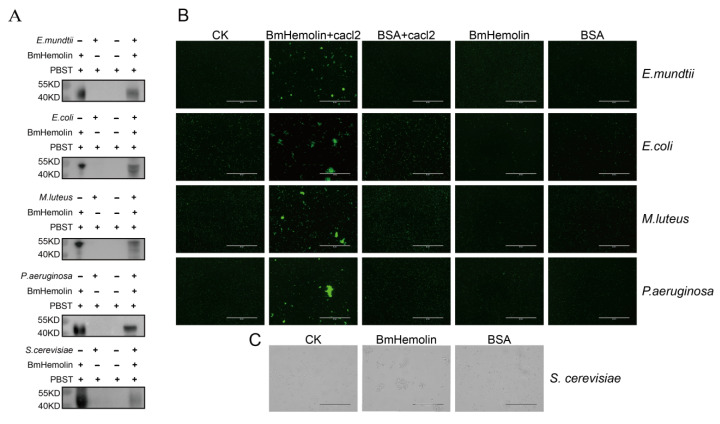
Binding and agglutination of recombinant BmHemolin protein with pathogenic microorganisms. (**A**) Western blot analysis of BmHemolin binding to pathogenic microorganisms. (**B**,**C**) The agglutination effects of BmHemolin with bacteria and fungi. Bacteria were treated with BmHemolin recombinant protein or BSA (as a negative control) in the presence or absence of Ca^2+^, while the fungus *S. cerevisiae* was treated only with BmHemolin recombinant protein or BSA (as a negative control). All treatments were compared with a blank control (CK) treated with Tris-HCl buffer.

To further explore the interaction between BmHemolin and pathogenic microorganisms, prokaryotic expression was performed to produce BmHemolin recombinant protein, which migrates at approximately 45 kDa. The target protein was purified by nickel column affinity chromatography and antibodies were prepared (Figure S1A–C). Subsequently, the binding and agglutination reactions of the BmHemolin recombinant protein with pathogenic microorganisms were detected. The BmHemolin recombinant protein was co-incubated with pathogenic microorganisms, and the incubated bacteria were washed with PBST. Western blot analysis revealed that the BmHemolin recombinant protein was detected in the bacterial precipitates of *E. mundtii, M. luteus, E. coli, P. aeruginosa, and S. cerevisiae* after washing, but not in the supernatant (Figure 3A). This indicates that BmHemolin can bind to these pathogenic microorganisms.

Further utilization of the agglutination reaction to detect the interaction between BmHemolin and these microorganisms. Results revealed that in the presence of calcium ions, the BmHemolin recombinant protein could induce agglutination of *E. mundtii*, *M. luteus*, *E. coli*, and *P. aeruginosa* (Figure 3B), whereas this phenomenon disappeared in the absence of calcium ions. The detection results of *S. cerevisiae* showed that BmHemolin also promotes the agglutination of *S. cerevisiae* (Figure 3C). The above results indicate that BmHemolin is involved in the binding and agglutination of various pathogenic microorganisms, thereby participating in the cellular immune response of silkworm.

### 3.4. BmHemolin Mediates Hemocyte Melanization and Exhibits Antibacterial Activity

**Figure 4:**
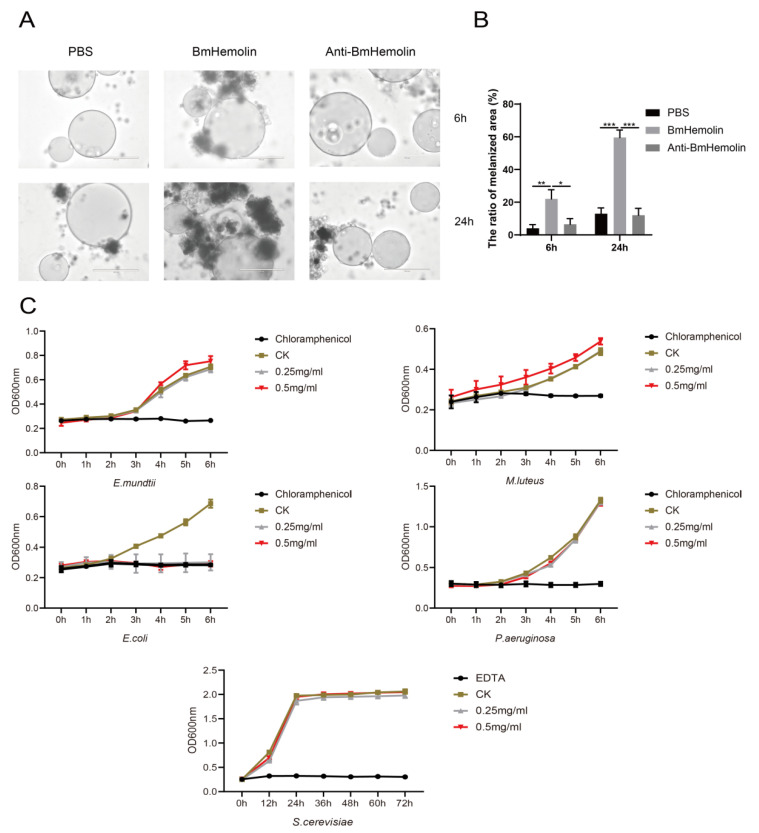
In vitro melanization assay and bacteriostatic test of BmHemolin. (**A**,**B**) observation and quantification of hemocytes melanization and aggregation. Error bars represent mean ± SD (*n* = 3), statistically significant differences are indicated as follows: * *p* < 0.05, ** *p* < 0.01, *** *p* < 0.001. (**C**) Antibacterial activity assay of BmHemolin. Chloramphenicol was used as the positive control for bacteria, and EDTA for fungi. Tris-HCl buffer served as the negative control (CK).

To further investigate whether BmHemolin mediates cell-mediated immunity involving hemocytes, in vitro experiments were conducted to observe and quantify the melanization and aggregation responses of hemocytes treated with BmHemolin recombinant protein. The results showed that Ni-NTA agarose beads without bound BmHemolin recombinant protein caused only a small number of hemocytes and melanization at 6 and 24 h. In contrast, beads bound with BmHemolin recombinant protein induced substantial hemocytes melanization and aggregation at the same time points (Figure 4A,B). These findings indicate that BmHemolin recombinant protein can participate in and promote the melanization and aggregation responses of hemocytes, phenomena that can be effectively blocked by BmHemolin antibody (Figure 4A,B).

To investigate whether BmHemolin can directly inhibit the growth of pathogenic microorganisms, an in vitro antibacterial assay of BmHemolin recombinant protein was conducted. The results show that BmHemolin recombinant protein can inhibit the growth of *E. coli* at both low (0.25 mg/mL) and high concentration (0.5 mg/mL) (Figure 4C), but has no significant inhibitory effect on the growth of *E. mundtii*, *M. luteus*, *P. aeruginosa*, and *S. cerevisiae* (Figure 4C). This indicates that BmHemolin can directly exert antibacterial effects as an immune effector molecule, but this is not its primary mode of immune action.

### 3.5. CRISPR/Cas9-Mediated Knockout of BmHemolin

**Figure 5:**
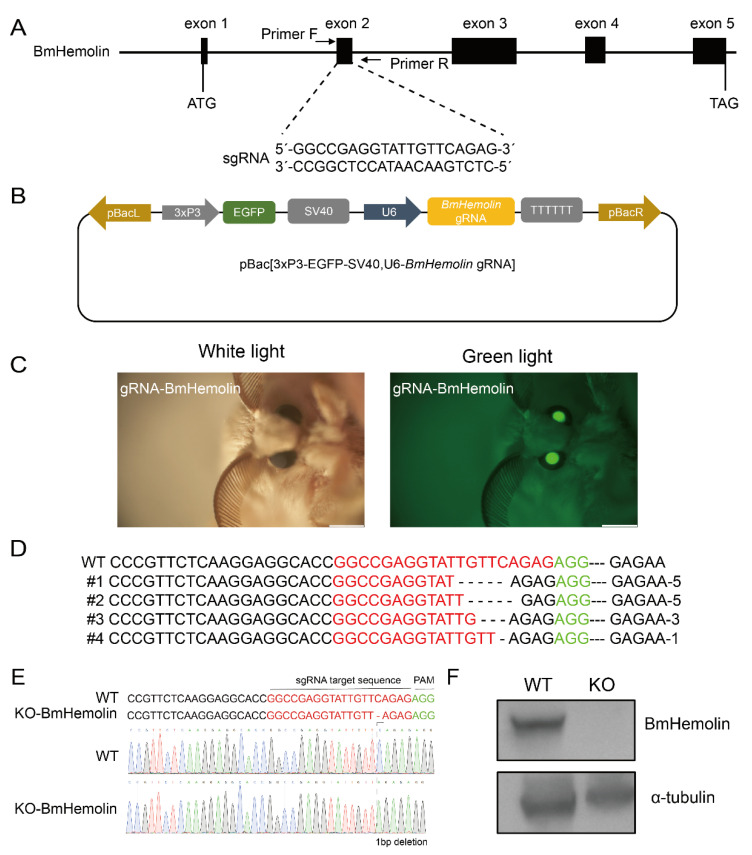
CRISPR/Cas9-mediated knockout of *BmHemolin*. (**A**) Schematic diagram of *BmHemolin*-gRNA location. (**B**) Schematic diagram of gene knockout vector. (**C**) Screening of positive individuals. (**D**) Analysis of gene editing patterns of G_2_ knockout individuals. (**E**) Genome sequencing of knockout individuals (*KO-BmHemolin*). (**F**) Western blot analysis of knockout individuals (*KO-BmHemolin*).

To investigate the roles of BmHemolin, we utilized CRISPR/Cas9-mediated genome editing to create a knockout of BmHemolin. Initially, a gRNA targeting the second exon of the BmHemolin gene was designed, and a pBac-based vector expressing BmHemolin-gRNA was developed (Figure 5A,B). Subsequently, this constructed vector was co-injected with the piggyBac helper plasmid into G_0_ silkworm eggs, allowing us to screen for offspring that exhibited green fluorescent eyes (Figure 5C). These individuals were then crossed with Cas9 transgenic individuals to produce the G_2_. The mutation types were detected, revealing different base deletion patterns in the G_2_ (Figure 5D). Further screening via Sanger sequencing, a *KO-BmHemolin* individual with a single-base deletion was identified (Figure 5E). The Western blot analysis results showed that BmHemolin protein was undetectable in *KO-BmHemolin* individuals, indicating that the *BmHemolin* gene was successfully knocked out (Figure 5F).

### 3.6. BmHemolin Enhances Microbial Clearance In Vivo

**Figure 6:**
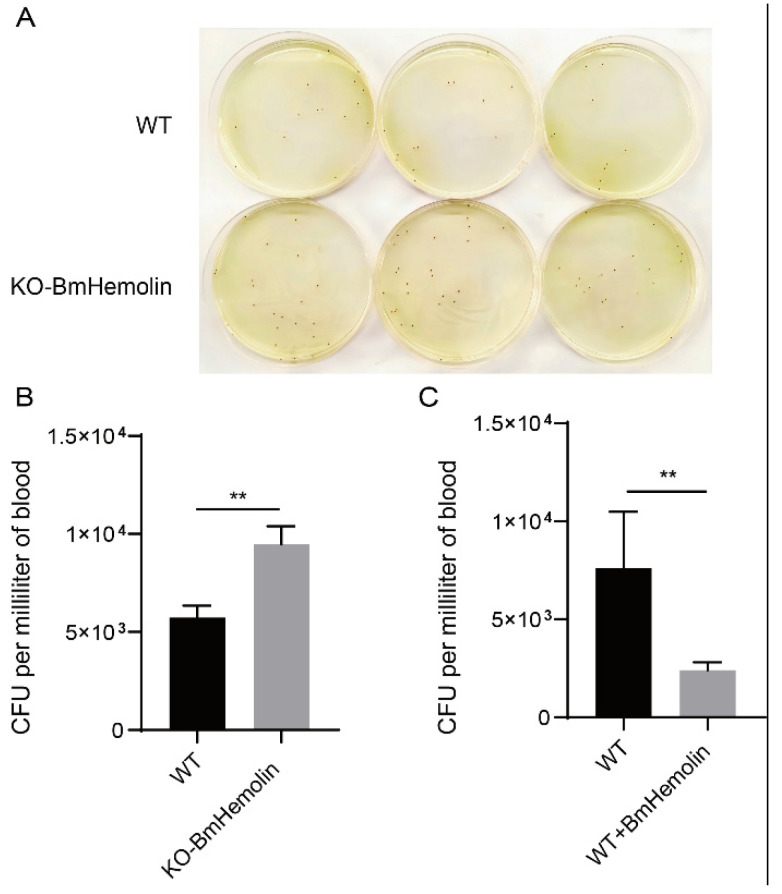
Microbial clearance assay in vivo. (**A**,**B**) Observation and quantification of recoverable *E. mundtii* in the hemolymph of WT and *KO-BmHemolin* silkworm larvae at 3 h post-injection. (**C**) Quantification of recoverable *E. mundtii* in the hemolymph of WT and Excessive BmHemolin (WT + BmHemolin) silkworm larvae at 3 h post-injection. Error bars represent mean ± SD (*n* = 3), statistically significant differences are indicated as follows: ** *p* < 0.01.

To investigate whether BmHemolin is involved in microbial clearance in vivo, we conducted *E. mundtii* injection experiments and found that *KO-BmHemolin* individuals exhibited significantly higher recoverable *E. mundtii* counts in their hemolymph compared to WT (Figure 6A,B). Furthermore, injection of BmHemolin recombinant protein resulted in a marked reduction in recoverable *E. mundtii* in the hemolymph (Figure 6C), demonstrating that BmHemolin participates in microbial clearance in vivo.

### 3.7. Humoral and Cellular Immunity Were Affected in BmHemolin Knockout Silkworms

To further investigate how BmHemolin participates in the immune response to pathogenic microorganisms, we conducted an in vivo *E. mundtii* injection experiment and examined the immune response of the silkworm. Compared with the injection of PBS, the gene expression of various AMPs, such as *Defensin2*, *Attacin1*, *Moricin2*, *CecropinD*, *CecropinB*, *Gloverin4*, and *Lebocin1/2*, was significantly upregulated 12 h after the injection (Figure S2A), In addition, the expression of genes related to cellular phagocytosis was also examined. The result showed that *Ced6*, *Actin A1*, and *TetraspainE* were significantly upregulated 12 h after injection (Figure S2B).

**Figure 7:**
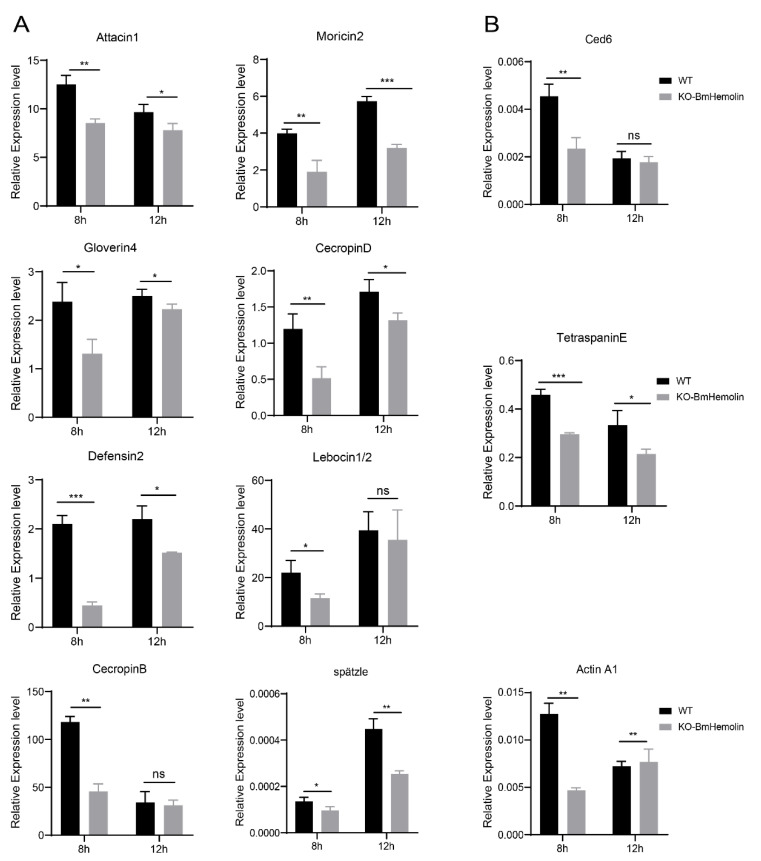
Effect of *BmHemolin* gene knockout on the expression of AMP genes in silkworms. (**A**) RT-qPCR analysis of AMPs and the key factor *spätzle* in the Toll pathway. (**B**) RT-qPCR detection of factors related to cellular phagocytosis. Error bars represent mean ± SD (*n* = 3), statistically significant differences are indicated as follows: * *p* < 0.05, ** *p* < 0.01, *** *p* < 0.001, ns indicates no significant difference.

Based on the aforementioned results, *E. mundtii* was injected into *KO*-*BmHemolin* and WT silkworm larvae. The results showed that the mRNA levels of AMP genes, including *Defensin2*, *Attacin1*, *Moricin2*, *CecropinD*, *CecropinB*, *Gloverin4*, and *Lebocin1/2* were significantly downregulated at 8 h post-injection in *KO-BmHemolin* individuals compared to the WT (Figure 7A). At 12 h post-injection, the expression of all AMPs except for *Lebocin1/2* and *CecropinB* remained significantly lower in the *KO-BmHemolin* individuals compared to the WT (Figure 7A). Further examination of *spätzle*, a key regulator of the Toll signaling pathway, revealed that the expression level of *spätzle* in *KO-BmHemolin* individuals was notably lower than in WT at 8 and 12 h post-injection (Figure 7A). These results suggest that BmHemolin may regulate the expression of AMPs through the Toll signaling pathway, thereby participating in humoral immunity.

Further, compared to WT, the mRNA levels of genes related to cellular phagocytosis were significantly downregulated in *KO-BmHemolin* individuals (Figure 7B). The above results indicate that BmHemolin is involved in the cellular immune response by promoting phagocytosis of hemocytes.

### 3.8. BmHemolin Can Enhance the Resistance of Silkworms to E. mundtii Infection

**Figure 8:**
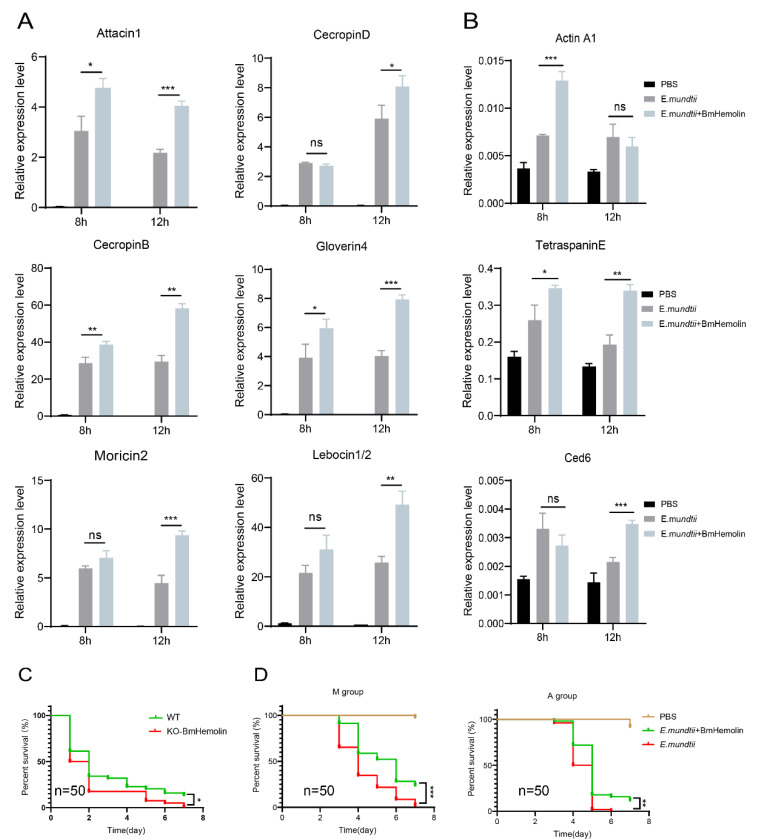
The impact of excessive BmHemolin on the immune defense of silkworms. (**A**,**B**) RT-qPCR analysis of AMP and phagocytosis-related genes. Error bars represent mean ± SD (*n* = 3), statistically significant differences are indicated as follows: * *p* < 0.05, ** *p* < 0.01, *** *p* < 0.001, ns indicates no significant difference. (**C**) Statistics on the survival rate of WT and *KO-BmHemolin* silkworm individuals after *E. mundtii* infection. Statistical analysis between the two groups was performed using the log-rank test (Mantel–Cox, *n* = 50). Statistically significant differences are indicated as follows: * *p* < 0.05. (**D**) Statistics chart of survival rate. M and A group: silkworms fed with mulberry leaves and artificial diet. Statistical analysis between experimental (*E. mundtii*+BmHemolin) and control *(E. mundtii*) groups were calculated by the log-rank test (Mantel–Cox, *n* = 50). Statistically significant differences are indicated as follows: ** *p* < 0.01, *** *p* < 0.001.

To investigate whether excessive BmHemolin could enhance the immune defense of silkworms against *E. mundtii*, BmHemolin recombinant protein was injected followed by injection of *E. mundtii* in vivo. The results showed that excessive BmHemolin could enhance the expression of various AMPs in silkworms, such as *Attacin1*, *Moricin2*, *CecropinD*, *CecropinB*, *Gloverin4*, and *Lebocin1/2* (Figure 8A), as well as the expression of phagocytosis-related genes *Ced6*, *Actin A1*, and *TetraspainE* (Figure 8B). Moreover, statistical analysis of the survival rates of silkworms indicated that the survival rate of *KO-BmHemlin* silkworms significantly decreased after infection with *E. mundtii* (Figure 8C). Injection of BmHemolin recombinant protein significantly increased the survival rate of silkworms fed with mulberry leaves and artificial diet after infection with *E. mundtii* (Figure 8D). The above results indicate that BmHemolin can enhance the immune resistance of silkworms to *E. mundtii*.

## 4. Discussion

Under normal conditions, the expression level of *Hemolin* in insects is relatively low, but it is significantly upregulated following infection by pathogenic microorganisms. Research by Daffre et al. found that Hemolin in *H. cecropia* is the only IgSF upregulated in response to infection [37]. This study further investigates how the upregulated BmHemolin participates in defending against infections by pathogenic microorganisms in silkworms.

The artificial diet rearing technology for silkworms has gradually emerged and been extensively studied. However, silkworms reared on artificial diets are in a sub-healthy state, showing maladaptation to the artificial diet and susceptibility to bacterial diseases caused by pathogenic microorganisms such as *E. mundti* [32,33]. Existing studies has focused on the adaptation of silkworms to artificial diets in terms of metabolic detoxification, with increased excretion by the Malpighian tubules and up-regulated expression of detoxification genes such as *GST*, *CYP*, and *UGT* [31]. However, studies on how silkworms respond to infections by pathogenic microorganisms such as *E. mundtii* and adapt to artificial diets in terms of immunity have not been reported as yet. In this study, we found that the immune gene *BmHemolin* was significantly upregulated in silkworms reared on artificial diet (Figure 1A–C), and it was further validated that pathogenic microorganisms such as *E. mundti* could significantly induce the upregulation of *BmHemolin* gene in the Malpighian tubules, Hemocytes, Fat body, and Midgut of silkworms (Figure 2A–G). This may explain the significant upregulation of BmHemolin expression in silkworms reared on artificial diet. The results indicate that BmHemolin plays an important immune role in silkworms reared on artificial diets.

Research has shown that PRRs participate in immune responses primarily by recognizing pathogens or danger signals, activating immune signaling pathways, and facilitating phagocytosis, thereby contributing to immune defense [38,39]. A minority of PRRs, such as PGRPs, can promote the agglutination of pathogenic microorganisms [40]. BmHemolin belongs to PRRs. This study found that its primary mode of action is to bind to various pathogenic microorganisms and promote their agglutination (Figure 3A–C). Its agglutination of bacteria requires the participation of calcium ions, whereas this is not necessary for fungi. It has not yet been determined how Hemolin binds to calcium ions. However, the literature reports indicate that Hemolin exhibits significant sequence similarity with the L1 family of transmembrane cell adhesion molecules (CAMs) [22]. Members of the L1 family mediate both homophilic and heterophilic adhesion events [41]. Hemolin, like related neural CAMs, exhibits homophilic adhesion properties and demonstrates calcium ion dependency [22,24]. Therefore, we hypothesize that after binding to bacterial surfaces, BmHemolin, in concert with calcium ions, promotes the aggregation of bacterial through its homophilic properties. Further, this study also found that BmHemolin participates in the immune system by enhancing the melanization and aggregation response of hemocytes, with this phenomenon becoming more pronounced over time. Within 24 h, compared to 6 h, the beads caused more melanization and aggregation of hemocytes (Figure 4A,B). Then, we discovered that BmHemolin can assist silkworm larvae in clearing *E. mundtii* from their bodies (Figure 6A–C). The above results indicate that BmHemolin can participate in defending against pathogenic microorganisms through cellular immunity and accelerate their clearance. It is noteworthy that the majority of PPRs do not possess the function of inhibiting the growth of pathogenic microorganisms, and there is no relevant evidence suggesting that Hemolin can inhibit the growth of pathogenic microorganisms. In this study, BmHemolin demonstrated a strong inhibitory effect on *E. coli* (Figure 4C), but it showed no growth inhibitory activity against other bacterial species such as *E. mundtii*, *M. luteus*, *P. aeruginosa*, and the fungus *S. cerevisiae* (Figure 4C). This result indicates that although BmHemolin has the capability to inhibit microbial growth, it is not the primary way it exerts its immune functions.

AMPs are a class of naturally occurring small molecular peptides produced by organisms, exhibiting broad-spectrum antimicrobial activity and the ability to modulate immune responses, making them an essential component of the innate immune system. [42,43,44]. So far, various AMPs have been identified, including antibacterial peptides, antifungal peptides, and antiviral peptides [45]. The relationship between Hemolin and AMPs was reported in a 2022 study by He et al., which demonstrated that interference with *ApHemolin* could reduce the expression of AMPs such as *Defensin*, *Attacin*, and *Moricin* [25]. In this study, the complete knockout of *BmHemolin* using CRISPR/Cas9 technology revealed that BmHemolin not only regulates the expression of *Defensin*, *Attacin*, and *Moricin* but also reduces the expression levels of AMPs such as *CecropinD*, *CecropinB*, *Gloverin4*, and *Lebocin1/2* (Figure 7A). Moreover, this regulatory effect was reversed when an excessive amount of BmHemolin was injected. The excessive BmHemolin induced an upregulation of AMP gene expression (Figure 8A), and this upregulation affected the survival rate of silkworms infected with *E. mundtii*, leading to an increase in their survival rate. This phenomenon was not influenced by the diet of the silkworms and was observed both in mulberry leaf-fed and artificial diet-fed conditions (Figure 8D). Conversely, the absence of BmHemolin significantly reduces the survival rate of silkworms infected with *E. mundtii* (Figure 8C). These results further confirm the regulatory role of Hemolin on AMP genes and suggest that the injection of BmHemolin may serve as a potential method to rescue silkworms infected with *E. mundtii* under artificial diet rearing conditions. In addition, in silkworms where *BmHemolin* was knocked out or injected with an excess of BmHemolin recombinant protein, we discovered the regulatory role of *BmHemolin* on genes related to hemocyte phagocytosis. Knockout led to the downregulation of hemocyte genes *Ced6*, *Actin A1*, and *TetraspaninE* (Figure 7B), while excess resulted in the upregulation of these genes (Figure 8B). Research has reported that *Ced6* is an adaptor protein involved in the phagocytosis of apoptotic cells [46], while *TetraspaninE* plays a significant role in pathogen recognition, endocytosis, transport, and interactions among immune cells [47,48,49,50]. This suggests that under pathogenic microorganisms’ stimulation, BmHemolin not only participates in immune resistance by regulating the expression of AMP genes but also influences cellular immunity through the regulation of phagocytosis-related gene expression. Previous studies have shown that Hemolin stimulates hemocytes through the activation of protein kinase C (PKC) and protein tyrosine phosphorylation pathways [51]. PKC and protein tyrosine phosphorylation precisely regulate phagocytosis through signal transduction, cytoskeletal reorganization, ROS generation, and phagosome maturation [52,53,54,55,56,57]. BmHemolin may regulate the expression of phagocytosis-related genes such as *Ced6*, *Actin A1*, and *TetraspaninE* by activating the PKC and protein tyrosine phosphorylation pathways.

Organisms regulate the production of AMPs through various immune pathways. In the humoral immunity of insects, there are four relatively important immune signaling pathways, namely the Toll, IMD, JAK/STAT, and JNK signaling pathways [1,58]. Among them, Gram-positive bacteria and fungi primarily activate the Toll pathway to produce AMPs [59,60], while Gram-negative bacteria and Plasmodium activate the IMD signaling pathway [61]. The JAK/STAT pathway is mainly involved in the immune response to Gram-negative bacteria and viruses [62]. Studies in *Drosophila melanogaster* showed that peptidoglycan from Gram-positive bacteria was identified by PPRs, which triggers the Toll pathway to promote AMP production [8]. In this study, the pathogenic bacterium *E. mundtii*, which is a Gram-positive bacterium, was used for stimulation. After *E. mundtii* stimulation, the detection of the key Toll signaling pathway factor *spätzle* in *KO-BmHemolin* silkworms revealed that the knockout of the *BmHemolin* gene significantly downregulated the expression of the *spätzle* gene (Figure 7A), This suggests that BmHemolin may activate the Toll pathway to express corresponding AMPs to defend against *E. mundtii*. Interestingly, although the Gram-positive bacterium *E. mundtii* was used as an inducer, the expression levels of AMPs regulated by the IMD pathway (such as *Attacin1*, *Moricin2*, and *Cecropin B/D*) also changed significantly (Figure 7A). This indicates that BmHemolin may simultaneously activate both the Toll and IMD signaling pathways. Additionally, existing studies have shown that there may be cross-activation or synergistic regulatory mechanisms between the Toll and IMD signaling pathways [6,58]. Pathogens may simultaneously activate multiple pathways or indirectly affect the expression of target genes in other pathways through shared signaling molecules [1,2]. BmHemolin is upregulated by pathogenic microorganisms, but it does not exhibit significant inhibitory effects on the growth of most pathogenic microorganisms. We speculate that BmHemolin primarily participates in the defense against pathogenic microorganisms indirectly by regulating cellular immunity and the expression of AMPs in humoral immunity.

## 5. Conclusions

This study discovered the significant upregulation of BmHemolin in silkworms reared on artificial diet. Subsequently, the reasons for its upregulation were analyzed, and further research was conducted on the mechanism by which BmHemolin participates in resisting the pathogenic microorganisms. The study revealed the crucial role of BmHemolin in silkworms’ defense against pathogenic microorganisms. It can bind pathogenic microorganisms and promote their agglutination, while also enhancing hemocyte melanization and agglutination. Additionally, it regulates the expression of AMPs and phagocytosis-related genes, thereby contributing to both cellular and humoral immunity in silkworms. In summary, Hemolin plays a pivotal role in both humoral and cellular immunity of insects. These findings have refined the role of the Hemolin gene in insect immune defense and offer guidance for better silkworm rearing on an artificial diet.

## Data Availability

The original contributions presented in this study are included in the article/Supplementary Materials. Further inquiries can be directed to the corresponding author.

## Supplementary Materials

The following supporting information can be downloaded at https://www.mdpi.com/article/10.3390/insects16080778/s1, Table S1: Primers used in this study. Figure S1: Purification of BmHemolin recombinant protein and antibody detection. Figure S2: Induction of antimicrobial peptide and phagocytosis gene expression in silkworms by *E. mundtii*. Figure S3: Original Western blot images for Figure 1C. Figure S4: Original Western blot images for Figure 2G. Figure S5: Original Western blot images for Figure 3A. Figure S6: Original Western blot images for Figure 5F. Figure S7: Original Western blot images for Figure S1C.
