# Supplementary material for: Functional Analysis of BmHemolin in the Immune Defense of Silkworms
Source: Insects. 2025 Jul 29;16(8):778. doi: 10.3390/insects16080778 (PMC12387071; doi:10.3390/insects16080778)
Supplement: Supplementary file 1 [file insects-16-00778-s001.zip › Figure S2.pdf]

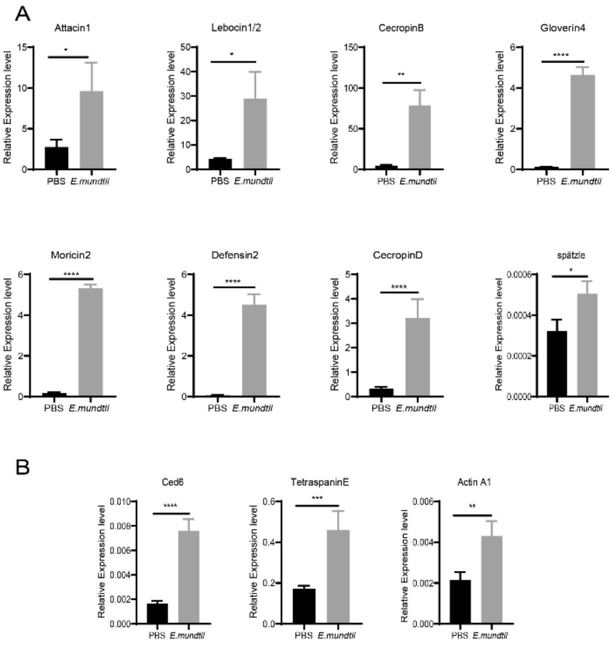

Figure. S2 Induction of antimicrobial peptide and phagocytosis gene expression in silkworms by *E. mundtii*. (A) RT-qPCR analysis of AMPs genes. (B) RT-qPCR analysis of phagocytosis gene. Error bars represent mean ± SD (n = 3), Statistically significant differences are as follows: *p<0.05, **p<0.01, ***p<0.001, ****p<0.0001.
