# Supplementary material for: Functional Analysis of BmHemolin in the Immune Defense of Silkworms
Source: Insects. 2025 Jul 29;16(8):778. doi: 10.3390/insects16080778 (PMC12387071; doi:10.3390/insects16080778)
Supplement: Supplementary file 1 [file insects-16-00778-s001.zip › Figure S3-Original Western blot images for Figure 1C.pdf]

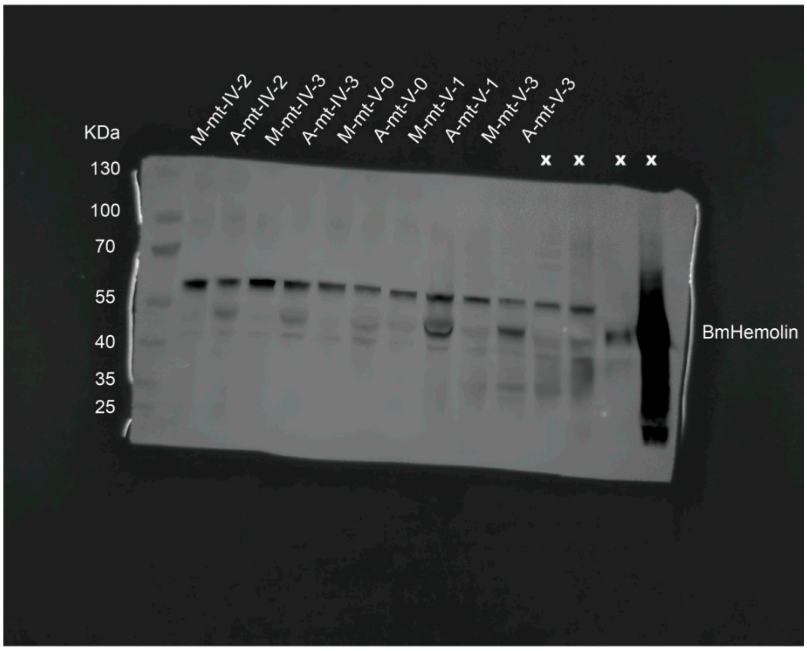

Expression profile analysis of BmHemolin in the Malpighian Tubules of silkworms fed with mulberry leaves (M-mt) and artificial diet (A-mt). IV-2: 2nd day of 4th insta; IV-3: 3rd day of 4th insta; V-0 to V-3: 0 h to 3rd day of 5th insta. X lane: the lane not used in the article.

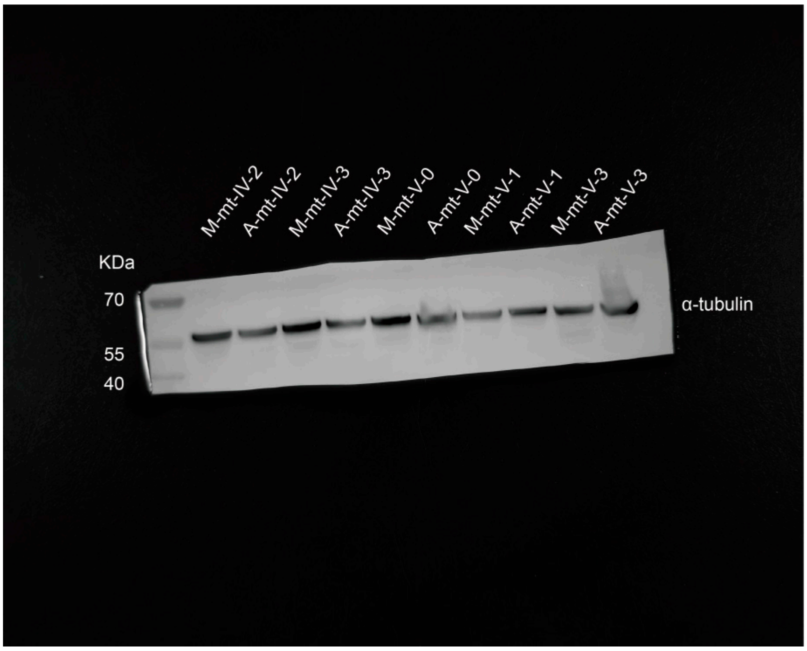

Expression profile analysis of α-tubulin in the Malpighian Tubules of silkworms fed with mulberry leaves (M-mt) and artificial diet (A-mt). IV-2: 2nd day of 4th insta; IV-3:3rd day of 4th insta; V-0 to-V-3: 0 h to 3rd day of 5th insta.

Sample	BmHemolin(mean)	α-tubulin(mean)	Ratio (BmHemolin/α-tubulin)
M-mt-IV-2	4.319	57.323	0.075345
A-mt-IV-2	29.623	47.682	0.621262
M-mt-IV-3	4.623	69.63	0.066394
A-mt-IV-3	30.341	42.887	0.707464
M-mt-V-0	4.314	69.16	0.062377
A-mt-V-0	27.589	66.646	0.413963
M-mt-V-1	16.186	35.404	0.45718
A-mt-V-1	96.251	50.5	1.90596
M-mt-V-3	15.951	49.616	0.321489
A-mt-V-3	61.152	60.326	1.013692

Quantitative Immunoblot Analysis of BmHemolin and α-tubulin in the Malpighian Tubules of silkworms fed with mulberry leaves (M-mt) and artificial diet (A-mt). IV-2: 2nd day of 4th insta; IV-3: 3rd day of 4th insta; V-0 to-V-3: 0 h to 3rd day of 5th insta. BmHemolin(mean): densitometric measurement of BmHemolin protein band intensity. α-tubulin(mean): densitometric measurement of α-tubulin protein band intensity.
