# Supplementary material for: Functional Analysis of BmHemolin in the Immune Defense of Silkworms
Source: Insects. 2025 Jul 29;16(8):778. doi: 10.3390/insects16080778 (PMC12387071; doi:10.3390/insects16080778)
Supplement: Supplementary file 1 [file insects-16-00778-s001.zip › Figure S4-Original Western blot images for Figure 2G.pdf]

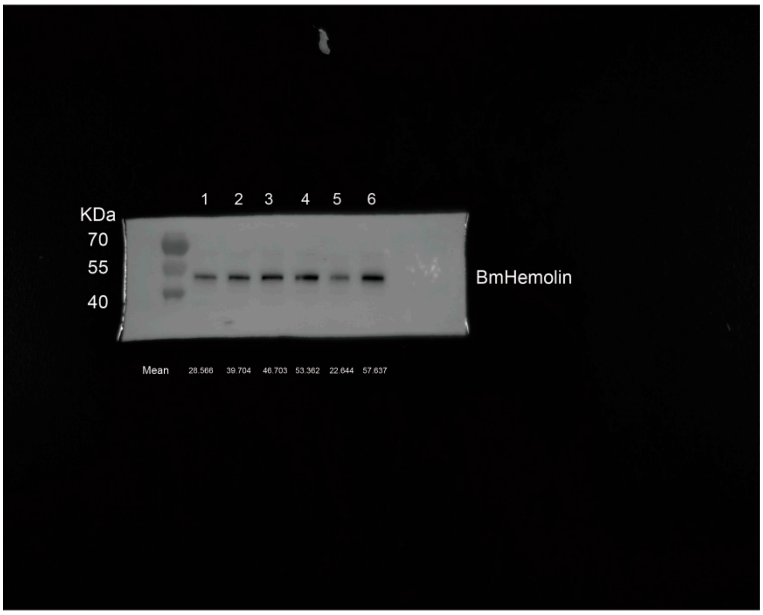

The changes of BmHemolin protein levels in the cell-free plasma after *E. mundtii* injection. Lanes 1, 3, 5: plasma of 5th instar larvae at 4, 8, and 12 h after PBS injection; Lanes 2, 4, 6: plasma of 5th instar larvae at 4, 8, and 12 h after *E. mundtii* injection. Mean: densitometric analysis of protein band intensity.

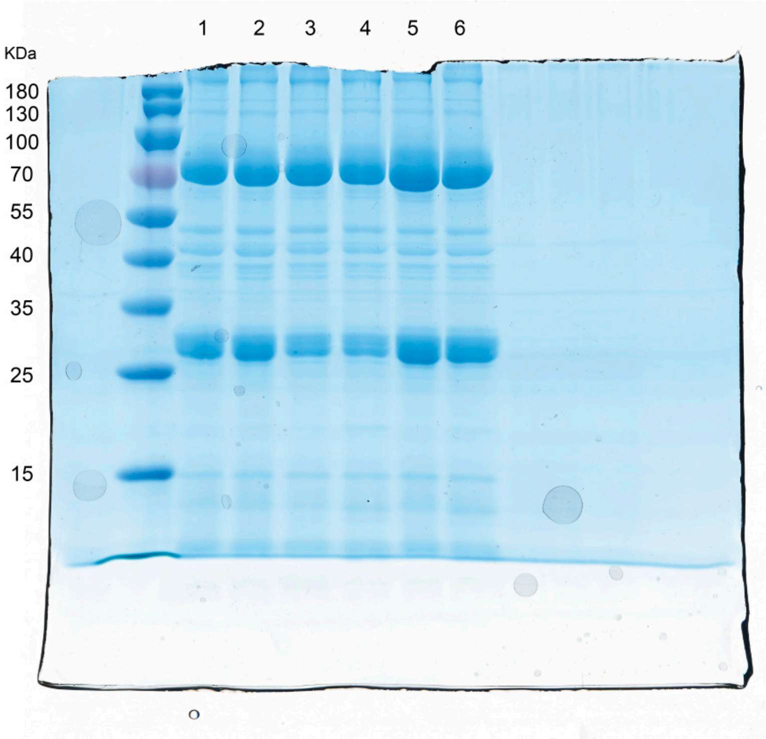

Ensuring consistent protein loading through Coomassie Brilliant Blue detection. Lanes 1, 3, 5: plasma of 5th instar larvae at 4, 8, and 12 h after PBS injection; Lanes 2, 4, 6: plasma of 5th instar larvae at 4, 8, and 12 h after *E. mundtii* injection.
