# Supplementary material for: Functional Analysis of BmHemolin in the Immune Defense of Silkworms
Source: Insects. 2025 Jul 29;16(8):778. doi: 10.3390/insects16080778 (PMC12387071; doi:10.3390/insects16080778)
Supplement: Supplementary file 1 [file insects-16-00778-s001.zip › Figure S5-Original Western blot images for Figure 3A.pdf]

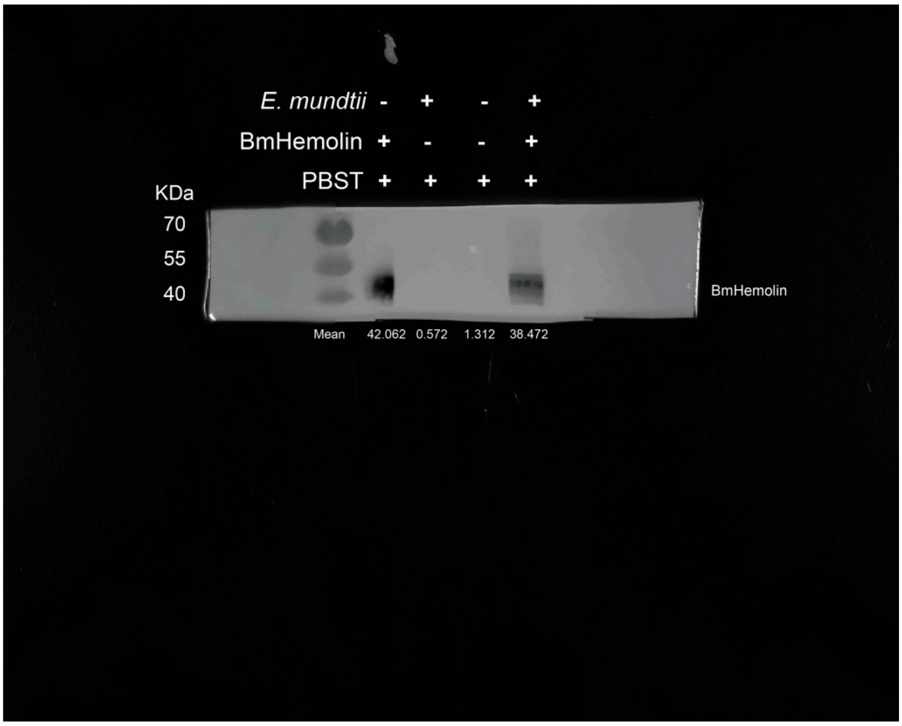

Western blot analysis of BmHemolin binding to *E. mundtii*. Mean: densitometric analysis of protein band intensity.

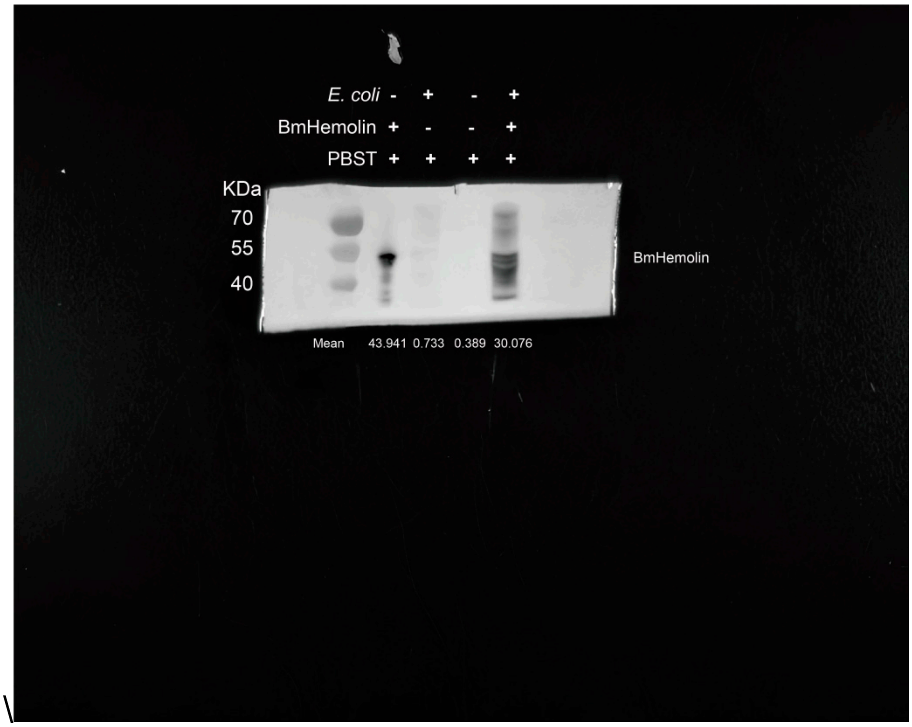

Western blot analysis of BmHemolin binding to *E. coli*. Mean: densitometric analysis of protein band intensity.

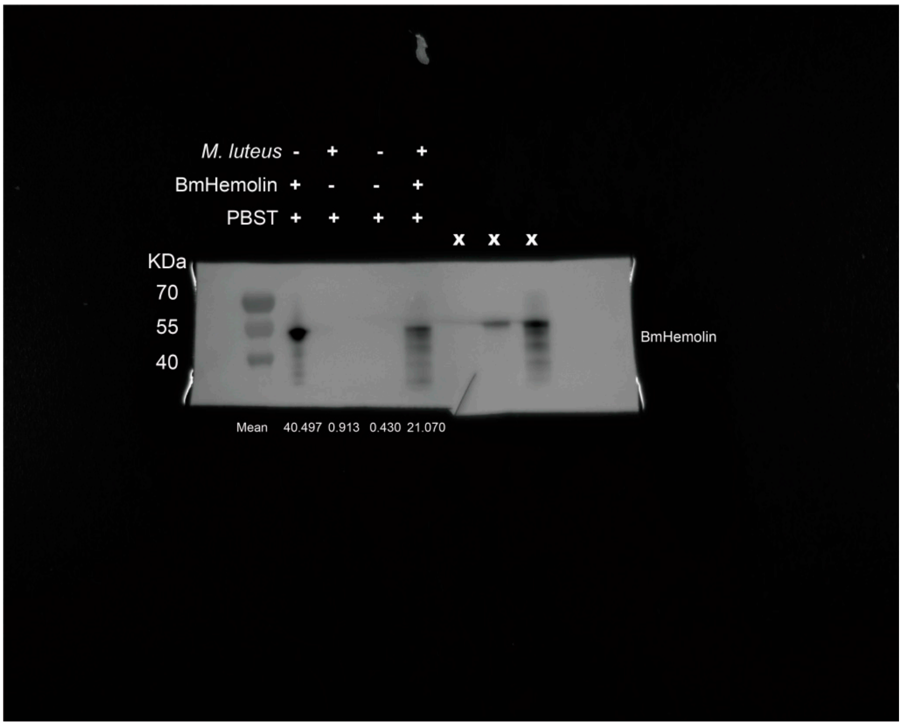

Western blot analysis of BmHemolin binding to *M. luteus*. Mean: densitometric analysis of protein band intensity. X lane: the lane not used in the article.

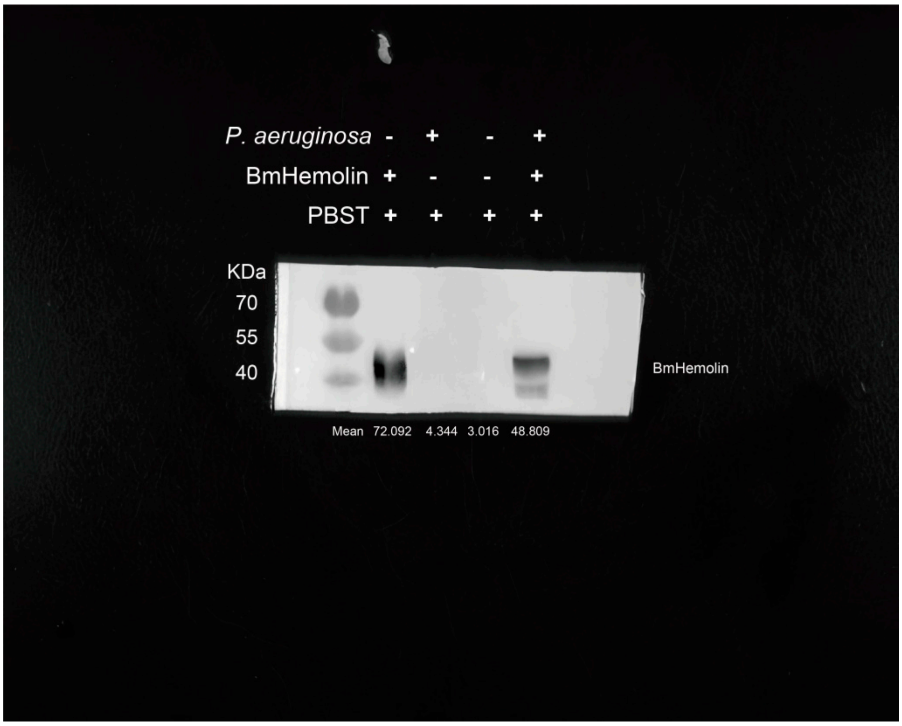

Western blot analysis of BmHemolin binding to *P. aeruginosa*. Mean: densitometric analysis of protein band intensity.

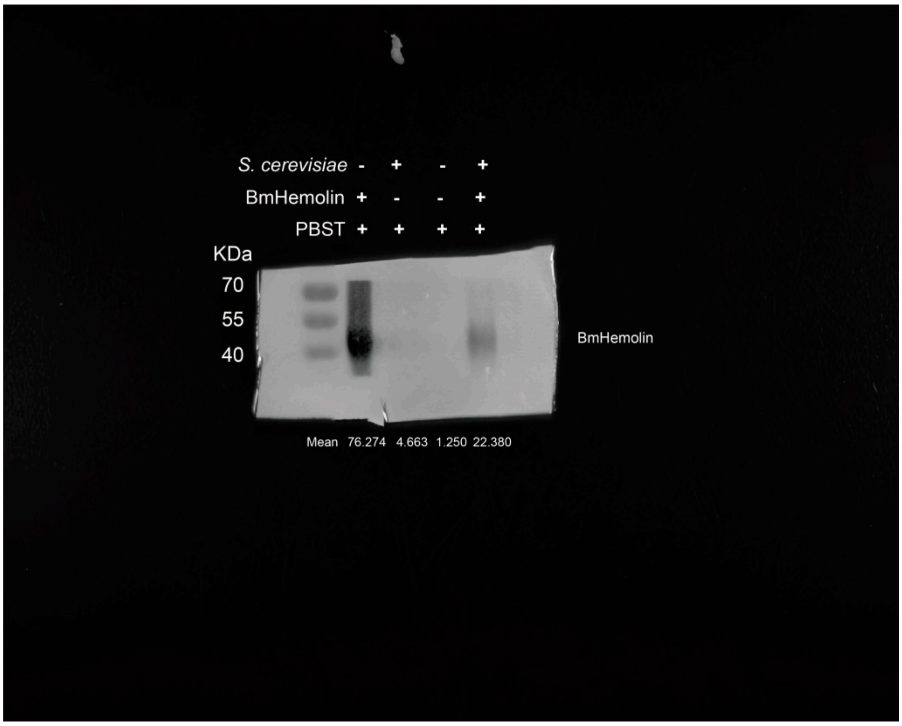

Western blot analysis of BmHemolin binding to *S. cerevisiae*. Mean: densitometric analysis of protein band intensity.
