# Supplementary material for: Functional Analysis of BmHemolin in the Immune Defense of Silkworms
Source: Insects. 2025 Jul 29;16(8):778. doi: 10.3390/insects16080778 (PMC12387071; doi:10.3390/insects16080778)
Supplement: Supplementary file 1 [file insects-16-00778-s001.zip › Figure-S6-Original Western blot images for Figure 5F.pdf]

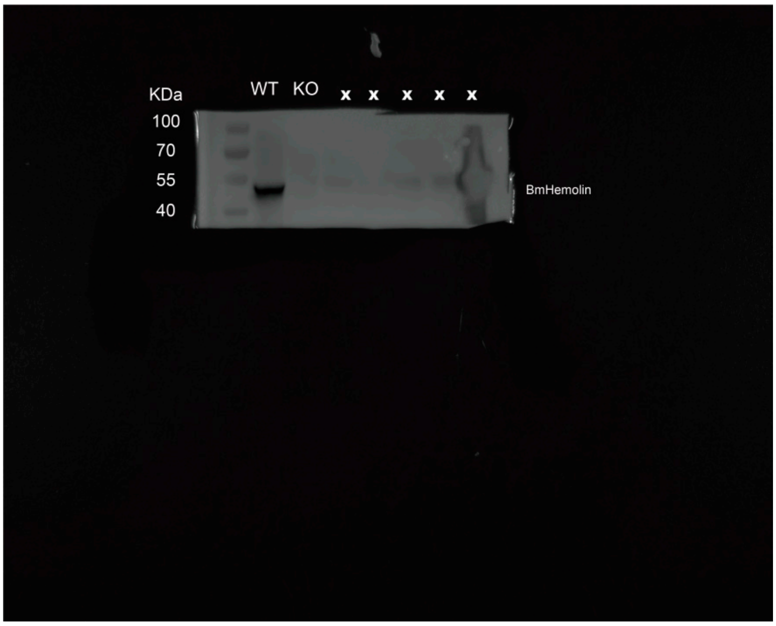

Western blot analysis of BmHemolin protein in knockout individuals (*KO-BmHemolin*) and WT individuals. X lane: the lane not used in the article.

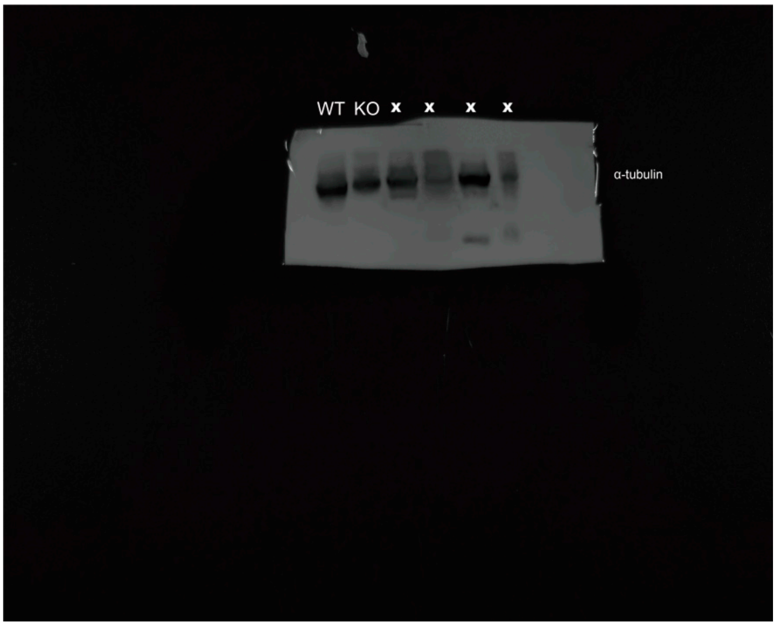

Western blot analysis of α-tubulin protein in knockout individuals (*KO-BmHemolin*) and WT individuals. X lane: the lane not used in the article.

Sample	BmHemolin(mean)	α-tubulin(mean)	Ratio (BmHemolin/α-tubulin)
KO	0.812	22.192	0.03659
WT	28.469	28.739	0.990605

Quantitative Immunoblot Analysis of BmHemolin and α-tubulin in knockout individuals (*KO-BmHemolin*) and WT individuals. BmHemolin(mean): densitometric measurement of BmHemolin protein band intensity. α-tubulin(mean): densitometric measurement of α-tubulin protein band intensity.
