# Supplementary material for: Functional Analysis of BmHemolin in the Immune Defense of Silkworms
Source: Insects. 2025 Jul 29;16(8):778. doi: 10.3390/insects16080778 (PMC12387071; doi:10.3390/insects16080778)
Supplement: Supplementary file 1 [file insects-16-00778-s001.zip › Figure-S7-Original Western blot images for Figure S1C.pdf]

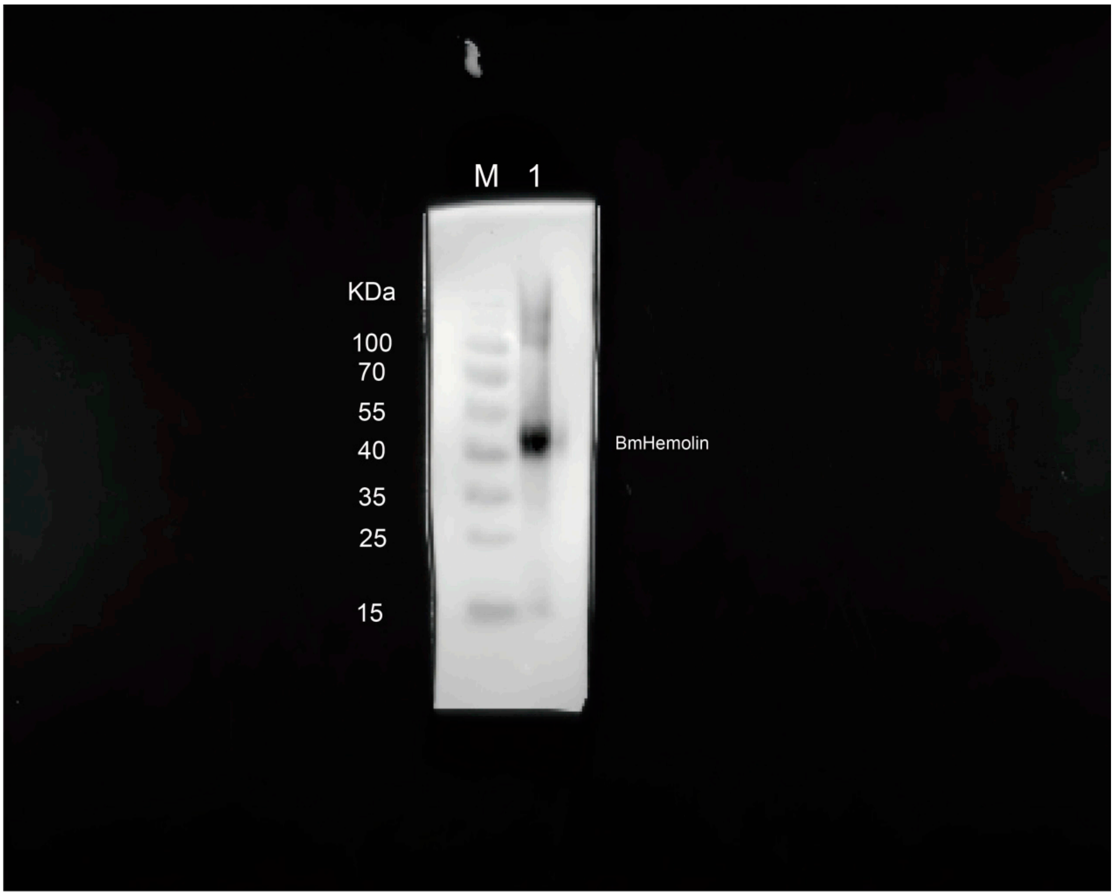

Western Blot Validation of BmHemolin recombinant protein. Lane 1: Purified BmHemolin recombinant protein.
