# Supplementary material for: Functional Analysis of BmHemolin in the Immune Defense of Silkworms
Source: Insects. 2025 Jul 29;16(8):778. doi: 10.3390/insects16080778 (PMC12387071; doi:10.3390/insects16080778)
Supplement: Supplementary file 1 [file insects-16-00778-s001.zip › Table S1.pdf]

**Table S1**. Primers uesed in this study

| Primer name | Primer sequence(5ˊ-3ˊ) |
|---|---|
| For protein experssion | |
| BmHemolin-F | CAGCCCGTTAATTCCGGAG |
| BmHemolin-R | TTAAGCGACTTGAAGAGCCG |
| For knockout gRNAs | |
| gRNA1-F | AAGTGGCCGAGGTATTGTTCAGAG |
| gRNA1-R | AAACCTCTGAACAATACCTCGGCC |
| gRNA2-F | AAGTGTCCCTCTCTGAACAATACCT |
| gRNA2-R | AAACAGGTATTGTTCAGAGAGGGAC |
| For RT-qPCR | |
| BmHemolin-F | GCATTTTAGTGTCGGTCAAGAC |
| BmHemolin-R | AATGCAGGAGTCTCTATGTACG |
| BmActin-F | TTCGTACTGGCTCTTCTCGT |
| BmActin-R | CAAAGTTGATAGCAATTCCCT |
| Gloverin4-F | AGGATATCCAATTAGCGGTCAG |
| Gloverin4-R | TGTAACCGGCTTTACCAAAAAG |
| Lebcoin1/2-F | CGTTTAACCCCAAGCCAATA |
| Lebcoin1/2-R | TGTCCCTCGGAATCAGAAAG |
| Moricin2-F | TGTGGCAATGTCTCTGGTGT |
| Moricin2-R | GCTTTCTTTTCTTCGGTTTCAA |
| Defensin2-F | ATACGTTGCTTCGACCTG |
| Defensin2-R | GAAACAGCCTTTGGGATT |
| CecropinB-F | CTATCCTTCGTCTTCGCTCT |
| CecropinB-R | ATAGCTTTAGCCGAACCGAG |
| CecropinD-F | CTCCGGCAACTTCTTCA |
| CecropinD-R | CGAACCCTCTGACCCATT |
| Attacin1-F | TTGTTGTGCGCGTGCTTG |
| Attacin1-R | CTTGTCGTTGCCGGTGAGAG |
| Spätzle-F | CAGGATTCGCCTCACAGTCAC |
| Spätzle-R | CAGTTCGGGATGCTTCCTCGAT |
| Ced6-F | GGACGGTGTTGCCATACAGG |
| Ced6-R | TTATCGTCCGCGCAGTACG |
| TetraspaninE-F | CAGCGTCCTCCTCTTCACCT |
| TetraspaninE-R | CCTCGTCTGCGTTAGCGTC |
| Actin A1-F | TCCTCCGTCTGGACTTGGC |
| Actin A1-R | CGATTTCCCTCTCAGCGGT |
